# Bacteriological Profile, Antibiotic Susceptibility Patterns, and Genotypic Characterization of Drug-Resistant Organisms in Chronic Suppurative Otitis Media at a Tertiary Care Hospital

**DOI:** 10.7759/cureus.112788

**Published:** 2026-07-16

**Authors:** Thilagavathi Govindhan, Therse Mary Dhason, Thyagarajan P Ravinder, Praveena V. G., K. Bhaskaran, Rayvathy Balasubramanian

**Affiliations:** 1 Microbiology, Arunai Medical College and Hospital, Tiruvannamalai, IND; 2 Microbiology, Bhaarath Medical College and Hospital, Chennai, IND; 3 Microbiology, Kilpauk Medical College, Chennai, IND; 4 Microbiology, Dr. ALM Post Graduate Institute of Basic Medical Sciences, University of Madras, Chennai, IND

**Keywords:** antibiotic resistance (abr), chronic suppurative otitis media (csom), esbl, methicillin resistant staphylococcus aureus (mrsa), pseudomonas-aeruginosa

## Abstract

Background

Chronic suppurative otitis media (CSOM) is a major cause of preventable hearing loss, particularly in developing countries. The emergence of multidrug-resistant pathogens has made empirical antibiotic therapy increasingly challenging. This study aimed to investigate the bacteriological profile, antimicrobial resistance pattern, and genotypic characteristics of drug-resistant bacterial isolates from patients with CSOM in a tertiary care setting.

Methodology

A hospital-based cross-sectional descriptive study was conducted among 111 patients with CSOM over one year. Ear discharge specimens were subjected to Gram staining, bacterial culture, biochemical identification, antimicrobial susceptibility testing using the Kirby-Bauer disc diffusion method (HiMedia Laboratories Pvt. Ltd., Mumbai, India) according to Clinical and Laboratory Standards Institute (CLSI), phenotypic detection of resistant organisms, and polymerase chain reaction (PCR) for *mecA *gene detection in methicillin-resistant *Staphylococcus aureus *(MRSA). Associations between culture positivity and demographic or clinical variables were analyzed using the Chi-square test.

Results

Among 111 participants, 68 (61.3%) were females, and 101 (91.0%) were adults. Culture positivity was observed in 90 (81.1%) patients, while 21 (18.9%) showed no bacterial growth. Gram-negative bacilli constituted 62 (68.9%) of isolates, with *Pseudomonas aeruginosa* being the predominant pathogen (50, 45.1%), followed by *S. aureus* (22, 19.8%) and *Klebsiella pneumoniae* (9, 8.1%). Meropenem demonstrated 100% sensitivity against *P. aeruginosa *and *K. pneumoniae*, while linezolid showed 100% sensitivity against *S. aureus*. MRSA accounted for 14/22 (63.6%) *S. aureus* isolates, and all MRSA isolates (14/14, 100%) were positive for the *mecA* gene by PCR, confirming genotypic methicillin resistance. Extended-spectrum β-lactamase (ESBL) production was detected in 12/59 (20.3%) Gram-negative isolates. Culture positivity was significantly associated with gender (*P* = 0.040) and age (*P* < 0.001) but not with complications (*P* = 0.617).

Conclusions

*P. aeruginosa* remains the predominant pathogen causing CSOM, with a substantial burden of multidrug resistance, including MRSA and ESBL-producing organisms. Routine culture, antimicrobial susceptibility testing, and molecular detection of resistance genes are essential for guiding appropriate therapy, optimizing antimicrobial stewardship, and improving clinical outcomes in CSOM.

## Introduction

Chronic suppurative otitis media (CSOM) is a persistent inflammatory disorder of the mucoperiosteal lining of the middle ear cleft, characterized by recurrent or continuous ear discharge through a perforated tympanic membrane and associated hearing impairment that persists for more than three to six weeks. This condition represents one of the most common infectious diseases of the ear, and if not treated appropriately, it may lead to serious complications, including facial nerve paralysis, lateral sinus thrombosis, labyrinthitis, and brain abscess [1]. CSOM remains a major public health concern, particularly in developing countries, where it is a leading cause of preventable hearing loss in children and contributes substantially to the social, economic, and healthcare burden on affected individuals, their families, and health systems [2]. The condition typically follows a multifactorial etiological pathway, often occurring as a sequela of recurrent acute otitis media or following upper respiratory viral infections, which are subsequently complicated by invasion of pyogenic organisms [2]. The incidence and prevalence of CSOM are influenced by several socio-environmental factors, including overcrowding, poor hygiene, inadequate nutrition, lack of health education, and chronic infectious diseases, all of which are more prevalent in resource-limited settings [2].

The microbiological profile of CSOM is diverse and includes both aerobic and anaerobic organisms as potential pathogens, though the reported profile and frequency of isolates vary considerably based on patient age, geographical region, and the presence of complications such as cholesteatoma [3]. Bacterial biofilms within the middle ear are recognized as an important reservoir of infection, facilitating bacterial persistence and contributing to the recurrent and chronic course of CSOM. The presence of these biofilms also reduces the effectiveness of antimicrobial therapy, making eradication of infection more difficult. Among the organisms isolated from the ear discharge of patients with CSOM, *Pseudomonas aeruginosa* is reported most frequently, followed by *Staphylococcus aureus*, *Klebsiella pneumoniae*, *Escherichia coli*, *Proteus *species, *Streptococcus* species, and coagulase-negative staphylococci. Polymicrobial infections are also commonly encountered in affected patients [3]. Anaerobic organisms, including *Bacteroides*, *Peptostreptococcus*, and *Propionibacterium* species, also contribute to the polymicrobial nature of CSOM. The presence of these diverse pathogens underscores the complexity of CSOM pathogenesis and highlights the importance of appropriate antimicrobial therapy [4].

CSOM continues to pose a significant therapeutic challenge because of its high prevalence, prolonged clinical course, increasing antimicrobial resistance, and the potential ototoxic effects associated with the empirical use of topical and systemic antibiotics. The inappropriate and excessive use of antimicrobial agents has accelerated the emergence of resistant bacterial strains, which contribute to both persistent disease and postoperative infections, ultimately compromising clinical outcomes. In recent years, multidrug-resistant organisms have become increasingly prevalent in patients with CSOM, with substantial resistance reported against several commonly prescribed antibiotics, including ampicillin, amoxicillin, co-trimoxazole, amoxicillin-clavulanate, cephalosporins, quinolones, and macrolides. This growing resistance has markedly reduced the effectiveness of conventional antimicrobial therapy and poses a major challenge to disease management [5]. The development of antimicrobial resistance in CSOM pathogens is particularly problematic as it limits therapeutic options, prolongs the course of infection, increases healthcare costs, and increases the risk of complications [4].

The antimicrobial susceptibility profile of pathogens associated with CSOM has been shown to vary across different geographic regions and over time, a trend that is largely attributed to the widespread and inappropriate use of antibiotics and differing prescribing practices across regions [5]. In vitro antibiotic susceptibility testing is essential for clinicians to plan an appropriate treatment regimen for patients with chronically discharging ears, as empirical therapy may be ineffective in the context of emerging resistance patterns [3]. Several studies have documented changing susceptibility patterns over time, with increasing resistance to previously effective first-line agents, underscoring the importance of regular surveillance of microbiological profiles and antimicrobial susceptibility patterns [6]. Molecular methods, including genotyping of drug-resistant organisms, have emerged as valuable tools for understanding the epidemiology of resistance and tracking the dissemination of resistance genes within populations [7]. These techniques can provide insights into the mechanisms underlying antimicrobial resistance and help guide infection control strategies [8].

The identification and characterization of resistant organisms through genotyping methods such as polymerase chain reaction (PCR) and sequencing of resistance genes have become increasingly important in the management of CSOM, particularly in the context of emerging multidrug-resistant strains. Understanding the genetic basis of resistance can aid in predicting susceptibility patterns, monitoring the spread of resistant clones, and developing targeted interventions to limit resistance dissemination [8]. The present study explored the current trend in bacteriological profile, antimicrobial susceptibility patterns, and genotypic characterization of drug-resistant organisms among CSOM patients attending a tertiary care hospital in Chennai. The findings of this study would provide valuable guidance for clinicians in selecting appropriate antimicrobial therapy, help formulate empirical treatment guidelines, and contribute to antimicrobial stewardship efforts in the management of CSOM.

## Materials and methods

Study design and setting

The present study was conducted in the Department of Microbiology at Government Tertiary Care Hospital, Chennai, a tertiary care teaching institution catering to a large urban and semi-urban population. The study was designed as a cross-sectional descriptive study and was undertaken during one year, extending from January 2020 through December 2020. The study included patients of both sexes who presented with chronic discharging ear and were attending the Ear, Nose, and Throat outpatient department or were admitted as inpatients during the study period. Aural swabs were collected from all eligible patients under aseptic precautions and processed for culture and sensitivity in the Microbiology laboratory. The study protocol was approved by the Institutional Ethical Committee (ECR/1385/Inst/TN/2020/431) before commencement, and written informed consent was obtained from all study participants. A structured questionnaire was used to collect relevant clinical and demographic data from each participant.

Sample size calculation

The sample size was estimated using the single population proportion formula based on an expected prevalence of 7.8% reported in a previous World Health Organization survey [9], with a 95% confidence level and a 5% margin of error. The formula used was *n* = *Z*²*P*(1 − *P*)/*d*², where *n* denotes the required sample size, *Z* is the standard normal deviate corresponding to the desired confidence level, *P* is the estimated prevalence, and *d* is the allowable margin of error. The minimum sample size calculated was 111 participants. Eligible patients were recruited consecutively using a convenience sampling approach until the required sample size of 111 participants was attained.

Inclusion and exclusion criteria

Patients of both male and female sexes across all age groups were included in the study if they had chronic ear discharge persisting for over 6 to 12 weeks, had central perforation of the tympanic membrane confirmed on otoscopic examination, and had not received prior antibiotics for the past one week. Patients who had received systemic or topical antibiotics for CSOM within the last 12 weeks were excluded from the study to avoid interference with culture results. Patients with a single and first episode of ear discharge were excluded, as were those with serious medical conditions such as diabetes mellitus, immune-compromised states, and malignancy, as these conditions could alter the bacteriological profile and influence antimicrobial susceptibility patterns.

Data collection

Demographic and clinical information were collected from all participants using a structured case record proforma. The recorded variables included patient identification details, age, sex, hospital registration number, date of pus sample collection, and a comprehensive history of the presenting illness. A thorough history was also obtained regarding past episodes of upper respiratory tract infections, acute otitis media, and any previous antibiotic usage. All collected data were entered into a predesigned case record form and maintained confidentially throughout the study period.

Sample collection

Aural specimens were collected from all study participants under strict aseptic conditions. Before sample collection, the external auditory canal was gently cleansed with sterile cotton soaked in 70% alcohol and allowed to air dry completely. Using adequate illumination and a sterile aural speculum, a sterile cotton-tipped swab was carefully inserted into the ear canal to obtain the purulent discharge while avoiding contact with the external auditory canal and surrounding skin to minimize contamination. Following collection, 0.5 mL of sterile normal saline was added to the transport container to prevent desiccation of the specimen during transport. Two swab specimens were obtained from the affected ear of each participant using the same standardized procedure, were labeled appropriately, and transported to the Microbiology laboratory as soon as possible for processing. The first swab was used for direct microscopy, including Gram staining, while the second swab was used for bacterial culture on appropriate media.

Direct microscopy

The first swab specimen was used to prepare a thin smear on a clean, grease-free glass slide for direct Gram staining. Microscopic examination of the stained smear was performed to assess the presence and quantity of pus cells, identify epithelial cells, determine the Gram reaction of the organisms (Gram-positive or Gram-negative), and evaluate their morphological characteristics, including cocci, bacilli, or coccobacilli. Gram staining was carried out according to standard microbiological procedures, using crystal violet as the primary stain followed by Gram's iodine as the mordant, decolorization with acetone, and counterstaining with dilute carbol fuchsin. The stained smear was then examined under an oil immersion objective of a binocular light microscope.

Bacterial culture

The second swab specimen was processed for bacterial culture by inoculating it onto nutrient agar, MacConkey agar, and 5% sheep blood agar plates. Nutrient agar served as a general-purpose medium for the isolation of a broad spectrum of bacterial organisms. MacConkey agar, a selective and differential medium containing peptone, sodium taurocholate, lactose, and neutral red indicator, was used to isolate Gram-negative bacilli and differentiate lactose-fermenting organisms from non-lactose fermenters. Blood agar, prepared with tryptose, beef extract, yeast extract, sodium chloride, agar, and 5% defibrinated sheep blood, was used to support the growth of fastidious organisms and to detect hemolytic patterns. All inoculated culture plates were incubated aerobically at 37 °C and examined for bacterial growth after 24 hours of incubation. Bacterial isolates were identified based on colony morphology, including size, shape, surface characteristics, edge, elevation, consistency, density, hemolysis on blood agar, and pigment production, followed by standard biochemical tests.

Biochemical Identification

Biochemical tests were performed on all bacterial isolates for definitive identification. The indole test was performed using peptone water containing sufficient tryptophan, with Kovac's reagent added after 48 hours of incubation, with the development of a red-colored ring in the alcohol layer indicating a positive reaction. The methyl red test was carried out using glucose phosphate peptone broth, and methyl red reagent was added after 48 hours of incubation. The appearance of a bright red color was interpreted as a positive methyl red reaction. The Voges-Proskauer test for acetoin production was performed by adding potassium hydroxide and alpha-naphthol solution to the glucose phosphate peptone water culture, with the development of a pink to crimson color indicating a positive reaction. Citrate utilization was tested on Simmons' citrate agar, where utilization of citrate was indicated by a blue color change of the bromothymol blue indicator. The urease test was performed on Christensen's medium, with the formation of a pink color indicating urease production. Catalase production was detected using 3% hydrogen peroxide, with the production of gas bubbles indicating a positive reaction. Oxidase activity was assessed using tetramethyl-p-phenylenediamine dihydrochloride reagent. The appearance of a deep purple coloration within 10 seconds was interpreted as a positive oxidase reaction. Triple sugar iron (TSI) agar was employed to differentiate enteric bacteria based on their ability to ferment glucose, lactose, and sucrose, as well as to detect hydrogen sulfide production. Coagulase testing was performed on staphylococcal isolates using both slide and tube methods; the slide test detected bound coagulase while the tube test detected free coagulase, with clumping or coagulum formation indicating a positive result.

Antimicrobial susceptibility testing

Antimicrobial susceptibility testing was performed for all bacterial isolates using the Kirby-Bauer disk diffusion method on Mueller-Hinton agar in accordance with the Clinical and Laboratory Standards Institute (CLSI) guidelines. Mueller-Hinton agar was used because of its excellent batch-to-batch reproducibility, ability to support the growth of most non-fastidious bacteria, and its recommendation as the standard medium for routine antimicrobial susceptibility testing. Pure colonies of each isolate were inoculated into peptone water broth and incubated at 35-37 °C for four to six hours. The bacterial suspension was then adjusted to the turbidity of a 0.5 McFarland standard (HiMedia Laboratories Pvt. Ltd., Mumbai, India), corresponding to an approximate inoculum density of 1.5 × 10⁸ CFU/mL. A uniform bacterial lawn was prepared by evenly swabbing the standardized inoculum over the surface of Mueller-Hinton agar (HiMedia Laboratories Pvt. Ltd., Mumbai, India using a sterile cotton swab. After allowing the inoculated plates to dry for 3-5 minutes, the appropriate antibiotic disks were placed on the agar surface using sterile forceps. The plates were subsequently incubated aerobically at 37 °C for 18-24 hours, after which the diameters of the zones of inhibition were measured in millimeters and interpreted according to CLSI breakpoints. Quality assurance was ensured by testing American Type Culture Collection (ATCC) reference strains, including *E. coli* ATCC 25922, *S. aureus* ATCC 25923, *K. pneumoniae* ATCC 700603, and *P. aeruginosa* ATCC 27853. The antimicrobial agents tested against Gram-negative bacilli included amikacin, gentamicin, ceftazidime, ciprofloxacin, meropenem, imipenem, and piperacillin-tazobactam, while the panel for Gram-positive cocci included penicillin G, cefoxitin, erythromycin, clindamycin, cotrimoxazole, ciprofloxacin, linezolid, and gentamicin, with interpretative zone diameters as per CLSI breakpoints.

Detection of carbapenemases

Phenotypic detection of carbapenemase production in Enterobacteriaceae and *P. aeruginosa* was performed using the CarbaNP test (bioMérieux, Marcy-l'Étoile, France) as a colorimetric microtube assay for epidemiological and infection control purposes. The test was performed by emulsifying a loopful of bacteria in bacterial protein extraction reagent in two labeled tubes, followed by the addition of solution A (without imipenem) and solution B (with imipenem) to the respective tubes. The tubes were incubated at 35 °C for up to 2 hours, and a color change from red to yellow indicated a positive result for carbapenemase production. Modified carbapenem inactivation method (mCIM) was also performed for the detection of carbapenemase production, where a 10 µg meropenem disk was incubated with the test organism in tryptic soy broth for four hours, and the inactivated disk was then placed on a Mueller-Hinton agar plate inoculated with a meropenem-susceptible *E. coli* ATCC 25922 indicator strain. Following overnight incubation, the zone of inhibition was measured, with reduced zone size indicating carbapenemase production.

Detection of MRSA

Methicillin resistance in *S. aureus* isolates was determined using the cefoxitin disk diffusion method (HiMedia Laboratories Pvt. Ltd., Mumbai) in accordance with CLSI recommendations. Morphologically similar colonies from pure culture were inoculated into broth, and the bacterial suspension was adjusted to match the turbidity of a 0.5 McFarland standard. A standardized lawn culture was prepared on Mueller-Hinton agar using a sterile swab, after which a 30-µg cefoxitin disk was placed on the inoculated agar surface. The plates were incubated at 35 °C for 16-18 hours under aerobic conditions. Following incubation, the diameter of the inhibition zone was measured and interpreted according to CLSI criteria. An inhibition zone of ≤21 mm was considered indicative of methicillin-resistant *Staphylococcus aureus* (MRSA), whereas a zone diameter of ≥22 mm was classified as methicillin-susceptible *Staphylococcus aureus* (MSSA).

Molecular identification of the *mecA* gene

The presence of the *mecA* gene, the principal determinant of methicillin resistance in *S. aureus*, was investigated by PCR following genomic DNA extraction using the QIAamp DNA Mini Kit (Qiagen GmbH, Hilden, Germany). Bacterial DNA was isolated using a commercial DNA extraction kit according to the manufacturer's protocol, which included enzymatic cell lysis with lysozyme, proteinase K digestion, DNA binding to silica spin columns, sequential washing, and final elution. The purity and integrity of the extracted DNA were assessed by agarose gel electrophoresis. PCR amplification of the *mecA* gene was carried out using a ready-to-use primer set comprising the forward primer 5′-GCAATCGCTAAAGAATAAG-3′ and the reverse primer 5′-GGGACCAACATAACCTAATA-3′, yielding an expected amplicon of 220 bp. The amplification protocol consisted of an initial denaturation at 95 °C for 5 minutes, followed by 35 cycles of denaturation at 94 °C for 30 seconds, annealing at 58 °C for 30 seconds, and extension at 72 °C for 30 seconds. A final extension step was performed at 72 °C for 5 minutes. The PCR products were subsequently analyzed by electrophoresis on a 2% agarose gel containing ethidium bromide and visualized under ultraviolet illumination to detect the expected amplification product, run at 50 V until the dye reached three-fourths of the gel distance, and visualized under a UV transilluminator. Bands were compared with a 100 base pair DNA ladder, and the presence of a 220 base pair band was considered positive for the *mecA* gene.

Statistical analysis

Data were entered and analyzed using IBM SPSS Statistics version 26.0 (IBM Corp., Armonk, NY). Categorical variables, including bacterial isolates, antimicrobial susceptibility patterns, and phenotypic and genotypic resistance profiles, were summarized as frequencies and percentages. Associations between categorical variables, such as antimicrobial resistance among different bacterial species and the agreement between phenotypic and genotypic methods for detecting antimicrobial resistance, were evaluated using the chi-square test or Fisher's exact test, where appropriate. A two-sided *P*-value of <0.05 was considered statistically significant. The study findings were presented using appropriate tables and figures to facilitate data interpretation.

## Results

Table 1 summarizes the demographic and clinical profiles of the 111 study participants. The largest proportion belonged to the 41-50 years age group (26, 23.4%), followed by 31-40 years (23, 20.7%) and 51-60 years (20, 18.0%). Adults constituted 101 (91.0%) participants, while paediatric patients accounted for 10 (9.0%). Females (68, 61.3%) outnumbered males (43, 38.7%). Complications requiring inpatient care were present in 38 (34.2%) participants, whereas 73 (65.8%) had uncomplicated disease. Unilateral CSOM was observed in 97 (87.4%) patients compared with bilateral disease in 14 (12.6%).

**Table 1:** Baseline demographic and clinical characteristics of study participants (N = 111).

Variable	Category	n	%
Age group (years)	10-20	13	11.7
	21-30	19	17.1
	31-40	23	20.7
	41-50	26	23.4
	51-60	20	18.0
	61-70	8	7.2
	71-80	2	1.8
Age category	Adult	101	91.0
	Pediatric	10	9.0
Gender	Female	68	61.3
	Male	43	38.7
Complications	Present	38	34.2
	Absent	73	65.8
Laterality	Unilateral	97	87.4
	Bilateral	14	12.6

Of the 111 clinical specimens, 90 (81.1%) yielded positive bacterial cultures, while 21 (18.9%) showed no growth. Among the culture-positive isolates, Gram-negative bacilli constituted 63 (70.0%) isolates, whereas Gram-positive cocci accounted for 27 (30.0%). *P. aeruginosa* was the predominant pathogen, isolated in 50 (45.1%) patients, followed by *S. aureus* (22, 19.8%) and *K. pneumoniae* (9, 8.1%). Less frequently isolated organisms included coagulase-negative *Staphylococcus* (3, 2.7%), *Proteus mirabilis* (2, 1.8%), *E. coli* (2, 1.8%), and *Enterococcus* species (2, 1.8%). Among the *S. aureus* isolates, 14 (63.6%) were identified as MRSA, all of which were mecA-positive. Additionally, 12 (20.3%) Gram-negative isolates were identified as ESBL producers, indicating the presence of clinically significant antimicrobial resistance among the isolates (Table 2).

**Table 2:** Culture positivity and bacteriological profile of CSOM (N = 111). ^†^Percentage calculated among Gram-negative isolates tested for ESBL production. MRSA, methicillin-resistant *Staphylococcus aureus*; ESBL, extended-spectrum β-lactamase; CSOM, chronic suppurative otitis media

Parameter	*n* (%)
Culture results	
Culture-positive	90 (81.1)
Culture-negative	21 (18.9)
Type of organisms isolated (*n* = 90)	
Gram-negative bacilli	63 (70.0)
Gram-positive cocci	27 (30.0)
Distribution of Isolates (*n* = 111)	
Pseudomonas aeruginosa	50 (45.1)
Staphylococcus aureus	22 (19.8)
Klebsiella pneumoniae	9 (8.1)
Coagulase-negative *Staphylococcus *(CONS)	3 (2.7)
Proteus mirabilis	2 (1.8)
Escherichia coli	2 (1.8)
*Enterococcus *spp.	2 (1.8)
Antimicrobial resistance pattern	
MRSA among *S. aureus* isolates (*n* = 22)	14 (63.6)
mecA-positive MRSA (*n* = 14)	14 (100.0)
ESBL-producing Gram-negative isolates (*n* = 59^†^)	12 (20.3)

Among 38 patients with complications, 32 (84.2%) were culture-positive, and 6 (15.8%) were culture-negative, whereas among 73 patients without complications, 58 (79.5%) were culture-positive and 15 (20.5%) were culture-negative. Culture positivity was not significantly associated with the presence of complications (*χ*² = 0.251, *P* = 0.617). With respect to gender, 39 of 43 males (90.7%) were culture-positive compared with 51 of 68 females (75.0%), while culture negativity was observed in 4 males (9.3%) and 17 females (25.0%). This difference was statistically significant (χ² = 4.224, *P* = 0.040), indicating a higher culture positivity rate among males. Culture positivity also demonstrated a significant association with age group (*P* < 0.001), with younger age groups exhibiting higher rates of organism isolation than older patients (Table 3).

**Table 3:** Association of culture positivity with demographic and clinical variables (N = 111). Chi-square/Fisher's exact test. ^*^P-value < 0.05 is statistically significant.

Variable	Culture-positive, *n* (%)	Culture-negative, *n* (%)	*χ*²-value	*P*-value
Complications				
Present	32 (84.2)	6 (15.8)	0.251	0.617
Absent	58 (79.5)	15 (20.5)		
Gender				
Female	51 (75.0)	17 (25.0)	4.224	0.040*
Male	39 (90.7)	4 (9.3)		
Age group				
<18 years	10 (100.0)	0 (0.0)	10.71	0.028*
≥18 years	80 (79.2)	21 (20.8)		

Among the 50 isolates of *P. aeruginosa*, all 50 (100.0%) were sensitive to meropenem. High susceptibility was observed for piperacillin-tazobactam and amikacin (47, 94.0% each), followed by gentamicin (44, 88.0%) and ceftazidime (38, 76.0%). Resistance was highest to amoxicillin-clavulanate and ceftriaxone, with 47 (94.0%) isolates resistant to each antibiotic, while 39 (78.0%) isolates were resistant to ofloxacin. Among the 9 isolates of *K. pneumoniae*, all 9 (100.0%) were sensitive to meropenem, piperacillin-tazobactam, ceftazidime, and gentamicin. Sensitivity to ceftriaxone and amikacin was observed in 7 (77.8%) and 6 (66.7%) isolates, respectively. The highest resistance was noted to ciprofloxacin, with 7 (77.8%) isolates resistant, followed by ampicillin with 5 (55.6%) resistant isolates (Table 4).

**Table 4:** Antibiotic susceptibility pattern of major Gram-negative isolates.

Antibiotic	*Pseudomonas aeruginosa* (*n *= 50)		*Klebsiella pneumoniae* (*n *= 9)	
	Sensitive, *n* (%)	Resistant, *n* (%)	Sensitive, *n* (%)	Resistant, *n* (%)
Meropenem	50 (100)	0 (0)	9 (100)	0 (0)
Piperacillin–Tazobactam	47 (94)	3 (6)	9 (100)	0 (0)
Amikacin	47 (94)	3 (6)	6 (66.7)	3 (33.3)
Gentamicin	44 (88)	6 (12)	9 (100)	0 (0)
Ceftazidime	38 (76)	12 (24)	9 (100)	0 (0)
Ceftriaxone	3 (6)	47 (94)	7 (77.8)	2 (22.2)
Ciprofloxacin/Ofloxacin	11 (22)	39 (78)	2 (22.2)	7 (77.8)
Amoxicillin–Clavulanate/Ampicillin	3 (6)	47 (94)	4 (44.4)	5 (55.6)

Among the 2 isolates of *P. mirabilis*, all 2 (100.0%) were sensitive to meropenem, piperacillin-tazobactam, amikacin, gentamicin, and ceftriaxone. One isolate (1, 50.0%) was sensitive to ciprofloxacin, whereas both isolates (2, 100.0%) were resistant to ceftazidime. The single *E. coli* isolate (*n* = 1) demonstrated 100% sensitivity to meropenem, piperacillin-tazobactam, amikacin, gentamicin, and ciprofloxacin (1, 100.0% each). However, it showed complete resistance to ampicillin and ceftazidime (1, 100.0% each).

Among the 22 isolates of *S. aureus*, all 22 (100.0%) were sensitive to linezolid. High sensitivity was also observed for erythromycin (20, 90.9%), whereas ciprofloxacin was effective against 12 (54.5%) isolates. Resistance was highest to ampicillin, with 20 (90.9%) resistant isolates, followed by cefoxitin, with 14 (63.6%) resistant isolates, indicating a high prevalence of methicillin resistance. Among the 4 isolates of coagulase-negative *Staphylococcus* (CONS), all 4 (100.0%) were sensitive to linezolid and clindamycin. Sensitivity to erythromycin and cefoxitin was observed in 2 (50.0%) isolates each, whereas all 4 (100.0%) isolates were resistant to ampicillin and ciprofloxacin. Among the 2 isolates of *Enterococcus* species, all 2 (100.0%) were sensitive to linezolid, vancomycin, and high-level gentamicin. In contrast, both isolates (2, 100.0%) were resistant to ampicillin and ciprofloxacin (Table 5).

**Table 5:** Antibiotic susceptibility pattern of Gram-positive isolates. CONS, coagulase-negative *Staphylococcus*

Antibiotic	*Staphylococcus aureus* (*n *= 22)		CONS (*n *= 4)		*Enterococcus* spp. (*n *= 2)	
	Sensitive,* n* (%)	Resistant,* n* (%)	Sensitive,* n* (%)	Resistant,* n* (%)	Sensitive,* n* (%)	Resistant,* n* (%)
Linezolid	22 (100)	0 (0)	4 (100)	0 (0)	2 (100)	0 (0)
Vancomycin	-	-	-	-	2 (100)	0 (0)
High-level Gentamicin	-	-	-	-	2 (100)	0 (0)
Clindamycin	20 (90.9)*	2 (9.1)	4 (100)	0 (0)	-	-
Erythromycin	20 (90.9)	2 (9.1)	2 (50)	2 (50)	-	-
Ciprofloxacin	12 (54.5)	10 (45.5)	0 (0)	4 (100)	0 (0)	2 (100)
Cefoxitin	8 (36.4)	14 (63.6)	2 (50)	2 (50)	-	-
Ampicillin	2 (9.1)	20 (90.9)	0 (0)	4 (100)	0 (0)	2 (100)

## Discussion

CSOM is a chronic inflammatory disease of the middle ear cleft that can lead to progressive tissue destruction and the development of serious complications if left untreated. The prolonged course of the disease plays a pivotal role in its progression, and infections are caused by a diverse range of both Gram-positive and Gram-negative bacterial pathogens [10]. The main medical management of CSOM includes topical antibiotics and aural toilet; hence, knowledge of the pathogen and its antibiotic susceptibility is mandatory before initiating therapy in CSOM. Approximately 111 study participants were enrolled, and specimens were collected to separate the organisms associated with CSOM and to identify the antibiotic susceptibility pattern of the bacteria causing the infection.

In this study, the majority of participants belonged to the adult age group, with the most affected age group being the fourth and fifth decades of life, which is consistent with the natural history of CSOM, where chronicity accumulates over years of recurrent infection. A higher proportion of affected patients were females compared to males, which is similar to the study conducted by Kaur et al., though most studies have shown higher prevalence among males [11]. This discrepancy may be attributed to differences in health-seeking behavior, as women may present more frequently to healthcare facilities, or to genetic factors, including disease resistance genes and their differential expression between sexes [12]. Nevertheless, there is insufficient evidence to establish a definitive association between patient sex and the bacteriological characteristics of CSOM.

Unilateral ear involvement in CSOM was found more commonly than bilateral involvement, which is similar to the study conducted by Kumar et al. [4], where approximately 80% of cases showed unilateral involvement, possibly due to poor hygiene and differential exposure. A similar report was also seen in a study conducted by Pawar et al., which showed unilateral involvement is more common than bilateral [13]. Though the reason for this predilection is not clearly established, many studies have reported that unilateral presentation has a better prognosis with limited hearing loss compared to bilateral disease. In the present study, a significant proportion of participants developed complications such as hearing impairment requiring hospital admission, which is in line with the study conducted by Loy et al. [12], who found that complications are more common in CSOM due to chronicity. The reasons for complications were found to be multifactorial, including chronicity, inappropriate medical treatment, delay in diagnosis, and negligence of patient self-care, similar to findings reported by Loy et al. [12].

The distribution of various organisms in specimens revealed that among culture-positive cases, among the culture-positive isolates, Gram-negative organisms predominated over Gram-positive organisms, which is similar to prior studies [14-16], where the presence of Gram-negative organisms was more common due to exposure to contaminated water and the ability of these organisms to survive in the hostile environment of the chronically discharging ear. A study conducted by Moorthy et al. also reported a similar pattern, concluding that while both Gram-positive and Gram-negative organisms cause middle ear infections, Gram-negative bacilli predominate in CSOM [16]. Among the positive culture growth, the majority were bacillary forms, with coccal forms comprising a smaller proportion. In this study, the predominant organism was *Pseudomonas* species, which is consistent with the findings of Vishwanath et al. [17], where *P. aeruginosa* was the most common isolate, followed by *S. aureus*. Another study conducted by Poorey et al. showed *Pseudomonas *species as the most common isolate organism in CSOM [2], with the incidence rate reported to vary from a significant proportion across different geographical regions. A study conducted by Keene et al. also demonstrated that *Pseudomonas* is the commonest organism responsible for CSOM [18]. The predominance of *P. aeruginosa* may be attributed to its numerous virulence factors, including the production of bacteriocins, expression of pili that facilitate adherence, and secretion of proteolytic enzymes, all of which enhance bacterial survival and persistence within the middle ear. In the present study, *S. aureus* was the second most frequently isolated organism, a finding consistent with that reported by Kumar et al., who also identified *S. aureus* as a major pathogen in CSOM [19]. The frequent isolation of *S. aureus* may be explained by its role as a commensal organism of the skin and anterior nares, from where it can opportunistically invade and establish infection under favorable conditions. A similar report was also found in a study conducted by Kaur et al., where *S. aureus* was isolated from a substantial proportion of patients [11]. The presence of variations in organisms is due to geographical changes, and the use of inappropriate antibiotics leads to gradual replacement by hardier and antibiotic-resistant organisms like coagulase-positive staphylococci, *Proteus *species, coliforms, and others in chronic infections.

Antimicrobial susceptibility testing was performed for all pathogenic isolates to determine their resistance profiles. Among *P. aeruginosa* isolates, carbapenems exhibited the highest level of susceptibility, followed by amikacin, piperacillin-tazobactam, gentamicin, and ceftazidime. In contrast, the greatest resistance was observed against amoxicillin-clavulanate and ceftriaxone. These findings are broadly consistent with those reported by Gulati et al., who identified amikacin as the most effective antimicrobial agent against *P. aeruginosa*, followed by ciprofloxacin, cefoperazone, gentamicin, cefotaxime, and amoxicillin [20]. The observed shift in susceptibility patterns compared with earlier reports reflects the evolving epidemiology of antimicrobial resistance, with previously effective agents such as streptomycin, tetracycline, and chloramphenicol demonstrating markedly reduced activity. Consequently, these antibiotics have largely been replaced in clinical practice by aminoglycosides, fluoroquinolones, and selected cephalosporins for the management of CSOM.

For *S. aureus* isolates, all were sensitive to linezolid, with high sensitivity to erythromycin and clindamycin, whereas high resistance was observed to ampicillin. This is similar to a study conducted by Indudharan et al., which reported that *S. aureus* was highly resistant to ampicillin [21]. The high prevalence of antimicrobial resistance observed in the present study may be explained by the referral pattern of patients to tertiary care hospitals, where many individuals seek treatment only after failure of initial empirical therapy. In addition, microbiological culture and susceptibility testing are often requested only for persistent or refractory infections, potentially introducing a selection bias toward more resistant pathogens. Resource constraints and high patient volumes in tertiary care settings may also limit the routine use of culture-guided therapy. In the present study, MRSA isolates outnumbered MSSA, a finding consistent with the observations of Pourmand et al. [22]. These findings emphasize the importance of early microbiological evaluation of ear discharge specimens, including pathogen identification and antimicrobial susceptibility testing, to facilitate appropriate targeted therapy and reduce the inappropriate use of broad-spectrum antibiotics that drives the emergence of resistant strains. Comparable findings were reported by Prakash et al., who also documented a high prevalence of multidrug-resistant organisms among patients with CSOM [23]. The increasing burden of antimicrobial resistance has been attributed to multiple factors, including inappropriate and indiscriminate antibiotic prescribing, over-the-counter availability of antimicrobial agents without prescription, inadequate microbiological diagnostic facilities, and deficiencies in antimicrobial stewardship and regulatory oversight. Collectively, these factors contribute to the growing challenge of antimicrobial resistance in developing countries, including India.

In the present study, the main gene involved in methicillin resistance among MRSA isolates was the *mecA* gene, identified by the PCR method. This is similar to a study conducted by Pourmand et al. [22], where among MRSA isolates, the majority were positive for the *mecA* gene identified by PCR, which is considered the gold standard method. A similar report was also found in a study conducted by Patigaroo et al., which concluded that MRSA is mainly acquired through either community-acquired, healthcare-associated, or nosocomial routes [15]. Methicillin resistance in *S. aureus* is primarily mediated by the *mecA* gene, which encodes the altered penicillin-binding protein PBP2a, conferring reduced affinity for β-lactam antibiotics and thereby rendering these agents ineffective. In addition to healthcare-associated strains, community-associated MRSA has emerged as a significant public health concern because of its increasing prevalence and ability to cause infections in otherwise healthy individuals, and its emergence in CSOM patients highlights the need for ongoing surveillance and antibiotic stewardship.

The present study has several notable strengths. The prospective design allowed systematic collection of specimens and comprehensive microbiological evaluation. The inclusion of molecular detection of the *mecA* gene provided definitive confirmation of methicillin resistance beyond phenotypic methods, enhancing the diagnostic accuracy. The study also evaluated a broad panel of antibiotics, providing a comprehensive antibiogram relevant to clinical practice.

However, several limitations must be acknowledged. The study was conducted at a single tertiary care center, which may limit the generalizability of findings to other settings, such as primary care facilities or community hospitals, where patient populations and antibiotic prescribing patterns may differ. The sample size, while adequate, may not capture the full spectrum of bacterial diversity seen in CSOM across different geographical regions. The study did not include anaerobic culture, which may have underestimated the contribution of anaerobic organisms to CSOM pathogenesis. The absence of longitudinal follow-up precluded assessment of clinical outcomes and the impact of antibiotic susceptibility results on treatment success. Additionally, molecular characterization beyond *mecA* gene detection, such as identification of other resistance genes, was not performed.

The findings of this study have several important clinical implications. The predominance of *P. aeruginosa* and *S. aureus* in CSOM underscores the importance of empirical antibiotic selection that covers these pathogens. The high resistance rates to commonly used antibiotics highlight the need for routine culture and sensitivity testing before initiating therapy. The presence of MRSA and the detection of the *mecA* gene reinforce the importance of appropriate antibiotic selection and the need for antimicrobial stewardship programs in healthcare facilities. Clinicians should be aware of the changing bacteriological profile and resistance patterns in their local settings and should consider these factors when selecting empirical antibiotics. The availability of rapid molecular methods for the detection of resistance genes can facilitate the timely institution of appropriate therapy and improve patient outcomes.

## Conclusions

In conclusion, this study demonstrates that Gram-negative organisms, particularly *P. aeruginosa*, are the predominant pathogens in CSOM, followed by *S. aureus*. The high prevalence of multidrug-resistant organisms, including MRSA, and the detection of the *mecA* gene highlight the urgent need for antimicrobial stewardship and routine culture and sensitivity testing before initiating therapy. The changing bacteriological profile and antibiotic susceptibility patterns over time emphasize the importance of continuous surveillance to guide empirical treatment and prevent the emergence of further resistance. Clinicians should be aware of local resistance patterns and should incorporate microbiological testing into the routine management of CSOM to ensure appropriate antibiotic selection and improve treatment outcomes.
